# An Activation Threshold Model for Response Inhibition

**DOI:** 10.1371/journal.pone.0169320

**Published:** 2017-01-13

**Authors:** Hayley J. MacDonald, Angus J. C. McMorland, Cathy M. Stinear, James P. Coxon, Winston D. Byblow

**Affiliations:** 1 Movement Neuroscience Laboratory, Department of Sport & Exercise Science, University of Auckland, Auckland, 1142, New Zealand; 2 Centre for Brain Research, University of Auckland, Auckland, 1142, New Zealand; 3 Clinical Neuroscience Laboratory, Department of Medicine, University of Auckland, Auckland, 1142, New Zealand; 4 School of Psychological Sciences and Monash Institute of Cognitive and Clinical Neurosciences, Monash University, Melbourne, 3800, Australia; Aston University, UNITED KINGDOM

## Abstract

Reactive response inhibition (RI) is the cancellation of a prepared response when it is no longer appropriate. Selectivity of RI can be examined by cueing the cancellation of one component of a prepared multi-component response. This substantially delays execution of other components. There is debate regarding whether this response delay is due to a selective neural mechanism. Here we propose a computational activation threshold model (ATM) and test it against a classical “horse-race” model using behavioural and neurophysiological data from partial RI experiments. The models comprise both facilitatory and inhibitory processes that compete upstream of motor output regions. Summary statistics (means and standard deviations) of predicted muscular and neurophysiological data were fit in both models to equivalent experimental measures by minimizing a Pearson Chi-square statistic. The ATM best captured behavioural and neurophysiological dynamics of partial RI. The ATM demonstrated that the observed modulation of corticomotor excitability during partial RI can be explained by nonselective inhibition of the prepared response. The inhibition raised the activation threshold to a level that could not be reached by the original response. This was necessarily followed by an additional phase of facilitation representing a secondary activation process in order to reach the new inhibition threshold and initiate the executed component of the response. The ATM offers a mechanistic description of the neural events underlying RI, in which partial movement cancellation results from a nonselective inhibitory event followed by subsequent initiation of a new response. The ATM provides a framework for considering and exploring the neuroanatomical constraints that underlie RI.

## Introduction

The most time-sensitive inhibitory responses in everyday life are commonly associated with unexpected events. Without foreknowledge of such events, cancellation of a response is difficult [1]. The cancellation of prepared movement when it is no longer appropriate is termed response inhibition (RI). A “horse-race” model provides a conceptual framework for RI [2]. This model posits that response execution results from neural facilitation triggered by an imperative cue, and that RI results from neuronal inhibition triggered by a stop signal. Whichever process “wins the race” to a point of no return somewhere along the neuroaxis determines whether or not a response is generated.

An anticipatory response inhibition (ARI) task conforms to the assumptions of the horse-race model with the added benefit of time-locking Go responses to a predictable event. In the ARI task Go responses can be anticipated and are therefore internally generated [3, 4], rather than made in response to an unpredictable imperative cue. The sudden cancellation of a single component of the prepared response can delay the remaining executed component by up to 100 ms [3, 5–7] or more in older adults [8, 9]. Interestingly, the response delay occurs regardless of whether the task requires dual responses made by different digits within the same hand (unimanual) or between hands (bimanual) [3]. While the traditional horse-race model can account for simple execution and inhibition in a RI task, we sought to determine whether or not it could account for the response delay during partial cancellation.

**Fig 1:**
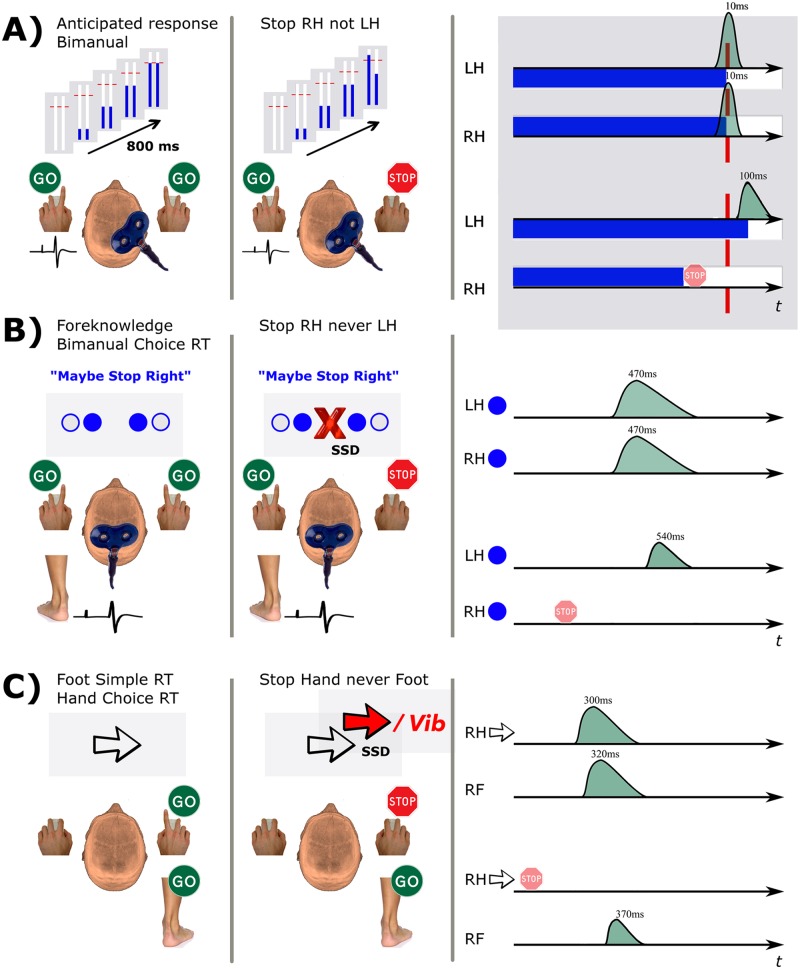
Experimental designs used to investigate the response delay. A: [7]. Bimanual anticipatory response inhibition task. On a Partial trial, one bar unexpectedly stopped rising before the target, cueing cancellation of the corresponding hand. TMS was applied to the right primary motor cortex and motor evoked potentials (MEPs) were recorded from the task-relevant left FDI muscle. Mean response times (ms) are relative to the target positioned at 800 ms. B: [13]. In the foreknowledge stop-signal task, text was displayed to bias stopping expectations followed two seconds later by the imperative. Participants made a synchronous bimanual choice reaction time (RT) response with both index or both little fingers. On Partial trials, a central stop signal appeared after a stop signal delay (SSD) and the stopping rule held in working memory was implemented. MEPs were recorded from the task-irrelevant tibialis anterior muscle. C: [15]. In the dual-task version of the stop-signal task, participants always responded with their foot and performed a choice RT task, responding with the hand that corresponded to the direction of the arrow (imperative). On Partial trials, the arrow turned red (or the hand was vibrated) after a SSD and no hand response was required. RTs are relative to the imperative in B and C. RH: right hand, LH: left hand, RF: right foot, RT: reaction time, Vib: vibration.

The response delay during partial RI has been confirmed across numerous studies performed by different groups [1, 3, 5–7, 10–14], even when the intention is to demonstrate that such delays can be minimised under certain conditions [15]. The delay appears robust to variations in the experimental paradigm e.g. whether the Go response requires reacting to an imperative or intercepting a stationary target. Here we will use the term response delay to reflect what others have called a stopping interference effect, or restart cost. The underlying mechanisms of response delays in the context of partial RI are not fully understood and have been explored with a variety of experimental paradigms, combining behavioural, neurophysiological and/or computational measures (Fig 1, [7, 13, 15]). These studies have investigated two important questions. What is the neural mechanism of the response delay observed during partial RI? And can a subset of a prepared multi-component response be selectively inhibited, with no delay in initiating the remaining response component?

Majid and colleagues [13] proposed a mechanism of selective inhibition and reduced response delays, but only when knowledge about stopping is given in advance. Majid and colleagues employed a variation of the traditional stop-signal task (SST) that required a bimanual choice reaction time (RT) in response to an imperative cue on the majority of trials. A visual stop signal presented after the imperative cued the cancellation of one component of the bimanual response. Using a dual-task SST, Xu and colleagues [15] suggested that selective inhibition is possible and the delay can be eliminated, even without foreknowledge. However, the study by Xu and colleagues showed robust delays across numerous conditions and minimal delays in a very specific condition: using a highly compatible tactile stop cue following relatively short stop signal delays (shorter than a standard simple reaction time), and only after multiple practice sessions in which a reward scheme de-emphasized accurate stopping. Combined these studies contend that partial RI is able to be implemented selectively at a neural level.

In contrast, we developed an activation threshold model (ATM) to account for response delays during partial RI [7] that proposed delays were inevitable due to non-selective inhibition following the stop cue. The ATM in this study was predicated on the findings that corticomotor excitability (CME) associated with the responding side was temporally modulated during partial RI in a manner that reflected anticipation, suppression and subsequent initiation of a reprogrammed response. The model proposes preparation of a synchronous two-component response via neuronal “coupling” of effector representations. An unanticipated stop signal triggers the simultaneous inhibition of all components of the prepared response and a new response comprised of a subset of the original effectors is initiated. The termination and reinitiation of movement accounts for the robust response delay.

The ATM predicts whether or not a response will occur, when it will occur, and with what gain, based on the net balance of facilitatory and inhibitory processes that compete upstream of motor output regions. Here we used a computational ATM, in conjunction with existing behavioural and neurophysiological data, to investigate hypotheses about the neural mechanisms of RI. A number of aspects expand this computational ATM beyond that proposed previously. Firstly, we validated and expanded the ATM by showing that optimized parameters of the model could reproduce experimental Go trial data distributions (as opposed to mean MEP and lift time modelled previously) to capture the variability of the facilitatory and inhibitory distributions inherent to Go trials of the ARI task. To date, the Go response distributions between the ARI and SST paradigms have not been directly compared. The computational ATM also allowed us to compare response distributions between different RI paradigms, by simulating empirical findings from the SST using results from [13]. We expected markedly different distributions of the underlying Go process [16, 17] between the ARI and SST, which may explain the opposing views in the literature regarding the selective nature of RI. The computational ATM also allowed us to further explore the hypothesis that response delays are due to nonselective response inhibition followed by response reprogramming. We expected that response delays could not be simulated solely by elevating an activation threshold via increased inhibition, thereby increasing the time to cross threshold (movement execution). Instead we expected an increase in threshold would necessarily be followed by an additional phase of facilitation representing a secondary activation process in order to cross the threshold and execute the remaining component of the response. Of note, the requirement for a secondary activation process was hypothesized in the previous ATM but not directly tested until now. Finally, we tested the computational ATM against a horse-race model (HRM) [2] to distinguish which was best able to capture behavioural and neurophysiological data during partial RI. We hypothesized that the ATM would produce a better fit than the HRM to data recorded during partial cancellation of movement on the ARI task.

## Methods

Empirical data for the ATM and HRM were taken from published work ([7]; Experiment 1). Data are from the left hand of 15 neurologically healthy right handed adults between the ages of 21–37 yr, 8 male. It was assumed that the empirical data came from a single normal distribution (healthy young adults) so the models were applied to aggregated group data. Participants completed the bimanual ARI task [3] while receiving single-pulse transcranial magnetic stimulation (TMS) of the right primary motor cortex (M1) over the first dorsal interosseous (FDI) hotspot (Fig 1A).

### Experimental procedure

#### Task

Participants were seated 1m in front of a computer display to perform the task. The task was controlled using custom software written in MATLAB (R2011a, version 7.12; The MathWorks) and interfaced with two custom-made switches, attached via an analog-to-digital USB interface (NI-DAQmx 9.7; National Instruments). The display consisted of two vertically oriented indicators 2 cm apart, 18 cm in length and 2 cm in width. The left indicator corresponded to the left index finger and the right indicator to the right index finger. Each trial commenced after a variable delay when both switches were depressed. Following the delay, both indicators moved upward from the bottom at equal rates and reached the target after 800 ms. The default Go response was bimanual index finger abduction to intercept the two rising indicators with the onscreen stationary target (Go trials). Visual feedback was displayed at the completion of each trial, indicating whether the indicator(s) had been stopped within 30 ms of the target, to emphasize that trials were to be performed as accurately as possible.

Occasionally one or both indicators stopped automatically before reaching the target (Stop trials), cueing the inhibition of the response with the corresponding digit(s). On Stop Both (Stop left—Stop right, SS) trials both indicators stopped automatically 600 ms into the trial and the participant was required to inhibit both fingers. Partial movement cancellation was required when only one indicator stopped (Partial trials), as participants were still required to intercept the other indicator with the target. The stop signal on Partial trials always occurred 250 ms prior to the target. The current models fit data from the left hand during successfully performed Go left—Stop right (GS) trials, when only the right hand is cued to stop and the response delay is seen in the left hand (Fig 1A, middle). A similar response delay occurs in Stop left—Go right trials but for simplicity we chose to model the nondominant hand in the first instance as it is more strongly affected by uncoupling during the bimanual ARI task [6]. The task was made up of of 12 blocks, each comprising 36 trials. Of the 432 trials in total, 288 (66%) were Go trials and 144 (33%) were Stop trials pseudorandomized across the 12 blocks. Typical lift time (LT) averages and distributions for this task are graphically depicted for Go (top) and GS trials (bottom) (Fig 1A, right).

#### Transcranial magnetic stimulation

Task motor threshold (TMT) was determined while the participant pressed the left switch as they would in the task. TMT was defined as the minimum stimulus intensity required to evoke FDI MEPs of at least 0.05mV amplitude in 4 of 8 stimuli. Test stimulus (TS) intensity was initially set at TMT and increased by 1–2% of maximum stimulator output if necessary to obtain a MEP amplitude of 0.1–0.2mV, without affecting behavioral performance. The TS intensity remained constant for the remaining data collection. TMS was delivered from 250–100 ms before the target in 25 ms intervals across 84 Go trials to obtain 12 stimuli at each of the seven times. To compare GS with Go trials, the 7 time points for single-pulse TMS were offset on GS trials by 100 ms, delivered from 150–0 ms prior to target, in 25 ms intervals (12 stimuli per stimulation time), because responses are delayed by about 100 ms on Partial trials [3, 6]. TMS was only ever delivered at a single time point on any one trial. Stimulation times were all pseudorandomized. There were 204 Go trials with no TMS interspersed throughout the blocks.

#### Recording procedure

Surface electromyography (EMG) was recorded from left FDI using a belly-tendon montage with the ground electrode placed on the posterior surface of the hand. The EMG collection system was triggered when the indicators started rising in the behavioral task, and EMG was recorded for 1 s.

### General model assumptions

In the current study, model parameters were estimated to fit the following experimental observations from [7]: i) an average response delay of 82 ms on Partial compared to Go trials; ii) a 50% decrease in CME on Partial compared to Go trials at equivalent time points preceding an EMG burst prior to the response; and iii) a higher eventual EMG burst gain on Partial versus Go trials to generate the response.

**Fig 2:**
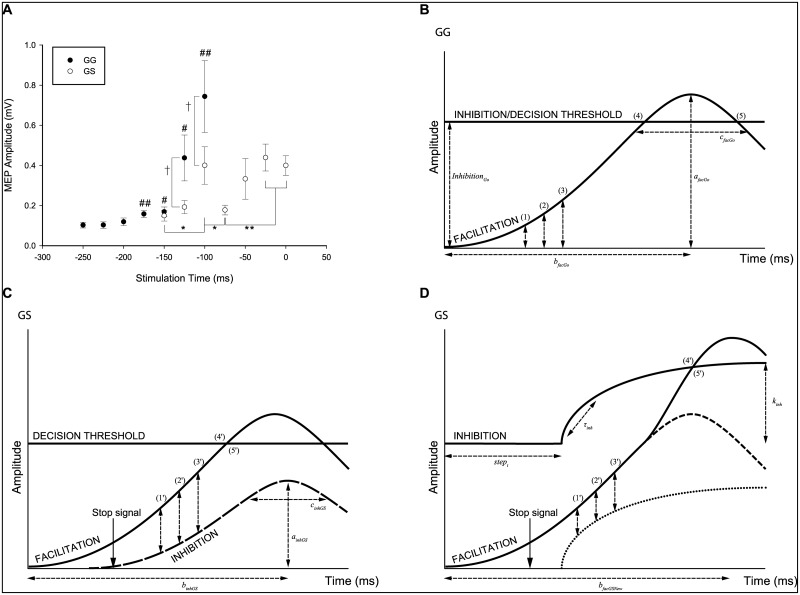
Experimental data used in the activation threshold and horse-race models. Graphic depiction of the experimental data from Go and GS trials in [7] and how the data is used in the activation threshold model (ATM) and horse-race model (HRM). A: Experimental results showing modulation of left first dorsal interosseous MEP amplitudes during Go (GG) and Partial (GS) trials. Stop signal was given at −250 ms on GS trials. Values are mean ± standard error. #*P* < 0.05; ##*P* < 0.001 represent significant increases relative to baseline during GG trials. †*P* = 0.052 denote trends. *P < 0.05; **P < 0.01 represent significant differences during GS trials. Reproduced from [7]. B: Model parameters for facilitation and inhibition curves in the ATM (equivalent Go and Stop processes in the HRM) were simultaneously fitted to motor evoked potential (MEP) amplitude data collected 150 (1), 125 (2) and 100 ms (3) before the target, and electromyography (EMG) onset (4) and offset (5) times. C: Model parameters for the Stop process on GS trials of the HRM were simultaneously fitted to MEP amplitudes 75 (1′), 50 (2′) and 25 ms (3′) before the target, as well as EMG onset times (4′) and rates of onset (5′). D: Model parameters for the increased inhibition and secondary facilitatory input were fitted to MEP amplitudes 75 (1′), 50 (2′) and 25 ms (3′) before the target, EMG onset times (4′) and rates of onset (5′). Note that the underlying facilitation process is equivalent for B–D which all illustrate the left hand response.

**Fig 3:**
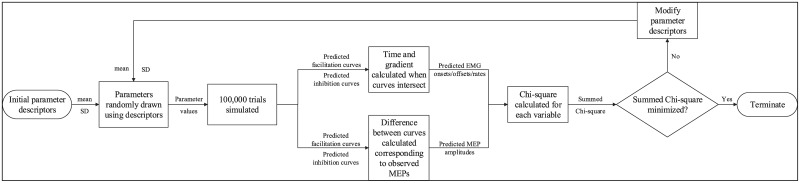
Model flowchart. Program flow for both models. Means and standard deviations (SD) were estimated for facilitatory and inhibitory function parameters to account for physiological variability. MEP: motor evoked potential; EMG: electromyography.

The models are specifically fitting the following experimental findings for Go trials (Fig 2B): i) the rise in average MEP amplitude from 150–100 ms prior to the target (150 ms: 0.17 ± 0.02mV, 125 ms: 0.44 ± 0.11mV, 100 ms: 0.74 ± 0.18mV); ii) average EMG onset time for trials stimulated 150–100 ms prior to the target (−50 ± 35 ms relative to target); and iii) average EMG burst duration for these same stimulated trials (107 ± 38 ms). For GS trials, the models are fitting (Fig 2C&2D): i) the dip in MEP amplitude 75 ms before the target (0.18 ± 0.03mV) and subsequent second rise in MEP amplitude 50 ms (0.33 ± 0.11mV) and 25 ms (0.44 ± 0.05mV) prior to the target; ii) average EMG onset time for trials stimulated 75–25 ms prior to the target (25 ± 46 ms after the target); and iii) an EMG burst onset rate 1.2 times higher than on Go trials (6.8 ± 1.2mVs^−1^ compared to 5.6 ± 1.1mVs^−1^). The models simultaneously fit entire distributions of MEP and EMG data (Fig 3).

**Fig 4:**
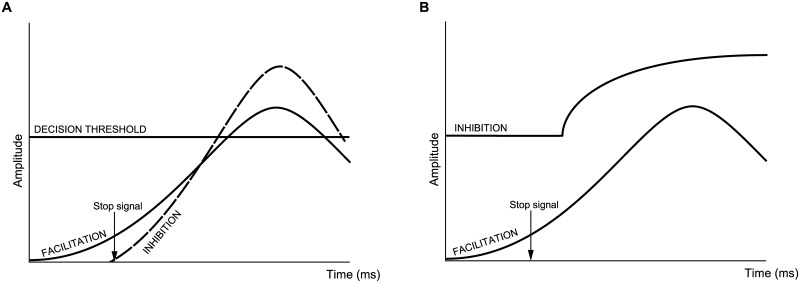
Theoretical comparison between model predictions for the suppressed finger on Partial trials. A: The horse-race model would predict that no response is generated when the Stop process reaches the decision threshold before the Go process. B: In contrast, the activation threshold model would predict that no response is generated when inhibition is raised to a level that cannot be reached by the preplanned Go response (facilitation curve). Both models predict the same behaviour resulting from distinct underlying mechanisms. Note that these mechanisms would also apply to both fingers on successful Stop Both trials when no response is generated with either finger.

MEP amplitudes recorded from FDI were used as a measure of the excitability of the corticomotor pathway in the lead up to the response. The MEP amplitude was equated with the difference between facilitation and the rise of inhibition above baseline (Fig 2C&2D). This reflects the subliminal fringe of neurons close enough to threshold to be activated by the TMS pulse, as a consequence of the balance of facilitatory and inhibitory input [18]. A simple Gaussian function was chosen to model facilitation leading to ballistic finger movements. Both models therefore assume between trial variability in the rate and starting point of CME accumulation reflecting an underlying ballistic facilitatory process. One distinction between models is the conceptualization of the threshold; a fixed point of no return (HRM), or tonic levels of inhibition (ATM) in the motor system. However the main distinction is the framework used to model inhibition on Partial trials (compare Fig 2C&2D), described below. This can also be seen in Fig 4 when comparing the model predictions for the suppressed finger on Partial trials (and indeed both fingers on successful Stop Both trials).

#### HRM

The Go and Stop processes reflect two independent pools of neural activity which increase towards a single threshold of neural activation (point of no return) somewhere along the neuroaxis. Whichever “wins the race” determines the behavioural outcome i.e. response execution or inhibition. The decision threshold is represented by a single value to denote a consistent location for this point of no return. Neural facilitation (the Go process) builds in anticipation to initiate the motor response and intercept the target on screen, and inhibition (the Stop process) builds in response to the stop signal to prevent motor output. The HRM assumes motor output is initiated once facilitatory input to the alpha motoneuron pool exceeds the threshold and marks the onset of the ballistic movement. Likewise if inhibitory drive reaches the threshold first it is assumed motor output and the ballistic movement is successfully prevented. It is worth noting that one process reaching the threshold does not preclude the other from continuing, as supported by evidence of MEP suppression [7] and force reduction [19] on unsuccessful Stop trials.

A simple Gaussian function, equivalent to that used for facilitatory drive, modelled the increased inhibition following the stop signal on GS trials (Fig 2C). Comparable underlying mechanisms were assumed for the Go and Stop process as the horse-race theory does not speak to a distinction between these processes.

#### ATM

The ATM is based on that proposed in [7]. The first version of the ATM in [7] was fit to the mean MEP and lift time data. In the present study the ATM has been fit to EMG onset times (as opposed to lift times) since these are more closely linked temporally to neural output. An assumption is that a response is not initiated until facilitatory inputs to the alpha motoneuron pool exceed inhibitory levels. In the lead up to a response, impulse control mechanisms [20–22] keep the motoneurons below threshold until a movement is appropriate. This framework is consistent with models of saccades whereby the growth of activity in neurons predicts saccade initiation time [23].

Increased inhibition following the stop signal was modelled with a step function including a time constant defining the rate of rise. Fig 2D illustrates the hypothesized increase in facilitatory drive required to pass the elevated levels of inhibition and lead to movement initiation.

### Model specifics

Predicted EMG and MEP data were generated by a Monte Carlo simulation reliant on a number of parameters describing the facilitatory and inhibitory processes hypothesized to underlie the movement response (Fig 3). Summary statistics (means and standard deviations) of predicted data were fitted to equivalent measures from experimental EMG and MEP distributions by minimizing a Pearson Chi-square statistic [24, 25] using the unconstrained Nelder-Mead simplex algorithm [26].

$$\begin{array}{c} \chi^{2}=\sum_{i}\sum_{j}{\left(o_{ij}-p_{ij}\right)}^{2}/p_{ij} \end{array}$$ (1)

where *i* indexes variables e.g. EMG onsets, MEP amplitudes, etc (Fig 2A&2B). Within each variable, experimental data points or model predictions were categorized into one of 3 bins, representing upper, middle and lower thirds, indexed by *j* such that *o*_*ij*_ and *p*_*ij*_ are observation and prediction counts respectively. Minimizing a summed Chi-square across all variables allowed simultaneous fitting of MEP amplitude and EMG data.

Parameter values from the original ATM [7] were initially passed into the optimization function. To ensure convergence on a global minimum, values from points on either side of estimated values were also compared. Facilitation and inhibition curves were determined across a time range spanning −400 to 200 ms relative to the target. A total of 100,000 trials were simulated with each set of parameters for all trial types. Given the time-intensive nature of the approach, simulations were run on a high-performance computing cluster operated by the University of Auckland Centre for e-Research as part of the New Zealand e-Science Infrastructure framework.

### Go trials

The modeling process for Go trials was the same between the HRM and ATM. MEP amplitudes and EMG data from Go trials were selected from all successful trials when TMS was delivered at 150, 125 and 100 ms prior to the target (N = 514). MEP amplitudes at these times depict the rise in CME above baseline prior to the lift response (Fig 2A). Facilitation curves were optimized to fit the observed MEP amplitudes. The decision threshold/level of inhibition was simultaneously optimized to create intersection points with the facilitation curves to fit the experimentally obtained EMG onset and offset times (Fig 2B).

To better capture the underlying neurophysiological processes associated with EMG onset/offset, the facilitation curve was modelled using a Gaussian base function (c.f., [7]). The simple Gaussian function was derived as

$$\begin{array}{c} Fac_{Go}=a_{facGo}e^{\left(-{\left(time-b_{facGo}\right)}^{2}/{2c_{facGo}}^{2}\right)} \end{array}$$ (2)

where *a*_*facGo*_ is amplitude, *b*_*facGo*_ is peak time, and *c*_*facGo*_ is curvature/width. As estimation of EMG offset times was noisy, offset times were generated based on the empirical average burst duration of 107 ms.

Means and standard deviations (SD) were estimated for each model parameter to account for: the speed of neuronal firing (curvature, *c*); the temporal and spatial summation of facilitatory inputs onto the alpha motoneuron pool (value at maximum, *a*); and the internal generation of an anticipated response to intersect the target (peak time, *b*). In the ATM, the activation threshold was set initially to reflect tonic inhibition in a resting state (*Inhib*_*Go*_). For each simulated trial, parameter values were drawn randomly from the normal distribution with the currently estimated mean and SD. The decision threshold was fixed in the HRM (*DecisionThresh*). Predicted EMG onset and offset times reflect the times the facilitation curves cross and recross the activation/decision threshold.

Since Gaussian curves have an infinite domain, a *minimum* value of 0.1 was set to calculate average time of onset (*t*_0_) for the facilitation curves. A minimum value of 0.1 was chosen to denote a rise in CME above resting level, and reflects empirical MEP amplitudes (in mV). The parameter values producing the best fit were entered into the following equation.

$$\begin{array}{c} t_{0}=\sqrt{-2c_{facGo}^{2}\log_{e}\left(threshold/a_{facGo}\right)}+b_{facGo} \end{array}$$ (3)

### Partial trials

TMS and EMG data were available from 258 successful GS trials, where the left hand was required to respond and intercept the indicator with the target but the right hand needed to be inhibited. Compared to Go trials, TMS times were delayed by 100 ms on GS trials to ensure TMS was delivered at equivalent time points to the behavioural response. The fitted stimulation times correspond to the suppression (-75 ms) and subsequent rise (−50 to −25 ms) in CME generating the delayed response in the left hand which was never cued to stop.

Time points −150 to −100 ms (Fig 2A) illustrate the initial rise in MEP amplitude on GS trials, demonstrating participants were starting to initiate a bimanual response prior to the stop signal. Therefore, optimized Go parameters generated the initial facilitation curves on GS trials. However the rise appears less steep on GS trials, indicating the inhibitory process is starting to have an effect.

#### HRM

The stop signal caused MEP suppression after 175 ms, conceptualized as initiation of the Stop process racing towards the decision threshold. The simple Gaussian function for inhibition was derived from Eq 2 using *a*_*inhGS*_, *b*_*inhGS*_, and *c*_*inhGS*_. The average time of onset (*t*_0_) for the inhibition curves was calculated using Eq 3.

#### ATM

The MEP suppression 175 ms after the stop signal was conceptualized as nonselective inhibition acting on the bimanual response to raise the activation threshold. The elevated threshold would need to be surpassed in the left hand for a response to occur and is a candidate mechanism to explain the inevitable response delay.

Inhibition increased in response to a step input with size *k*_*inh*_ and time constant *τ*_*inh*_

$$\begin{array}{c} Inhib_{GS}=Inhib_{Go}+k_{inh}\left(1-e^{-(time+step_{t})/\tau_{inh}}\right) \end{array}$$ (4)

where *step*_*t*_ captured temporal variability in stop signal processing.

A second rise in CME is seen empirically in order to generate the unimanual (left hand) response (Fig 2A). It was assumed that the neural facilitation mechanisms would be comparable to the initial bimanual response i.e., *a*_*facGo*_ = *a*_*facGSNew*_ and *c*_*facGo*_ = *c*_*facGSNew*_. Mean and SD for *b*_*facGSNew*_ were estimated for these secondary facilitation curves (Eq 5). Facilitatory inputs for the unimanual response were modelled as additive to the pre-existing bimanual facilitation at the level of the alpha motoneuron pool.

$$\begin{array}{c} Fac_{GSNew}=Fac_{Go}+a_{facGSNew}e^{\left(-{\left(time-b_{facGSNew}\right)}^{2}/{2c_{facGSNew}}^{2}\right)} \end{array}$$ (5)

The resulting unimanual response is produced at a higher gain than the original (bimanual) response, and this is evident in an elevated rate of EMG onset [6, 7] and greater force [12]. This increased gain of the motor system was captured by the slope at the point of intersection with the activation threshold and has been estimated as 120% of Go trials based on EMG data of [7].

### Go trials in the stop-signal task

To compare bimanual Go distributions between the SST and ARI task, the ATM generated facilitation curves based on the RTs from ([13], Experiment 2). For simplicity, RTs were transformed to the time scale used for the ARI task (SST go signal = −400 ms). Insufficient data existed to model Partial trials for the SST.

It was assumed that the fundamental neural characteristics of ballistic responses in the ARI task are comparable to the SST. Therefore optimized ARI values were used for *a*_*facGoSST*_, *c*_*facGoSST*_ and *Inhib*_*GoSST*_. The mean and SD for *b*_*facGoSST*_ were estimated based on the empirical RTs. The fitting process included the same steps, with the following exceptions:

1. Given that EMG onsets were not measured, we assumed a consistent electromechanical delay and the ATM parameters were optimized to match curve intersection points to behavioural RTs. Therefore the comparison of distribution patterns, but not absolute *b* values, is valid between tasks.
2. Experimental RT data were generated by randomly sampling from a right-skewed [17, 27] skew normal distribution with a mean of 472 ms and SD of 49 ms [13].

## Results

### Go trials

**Fig 5:**
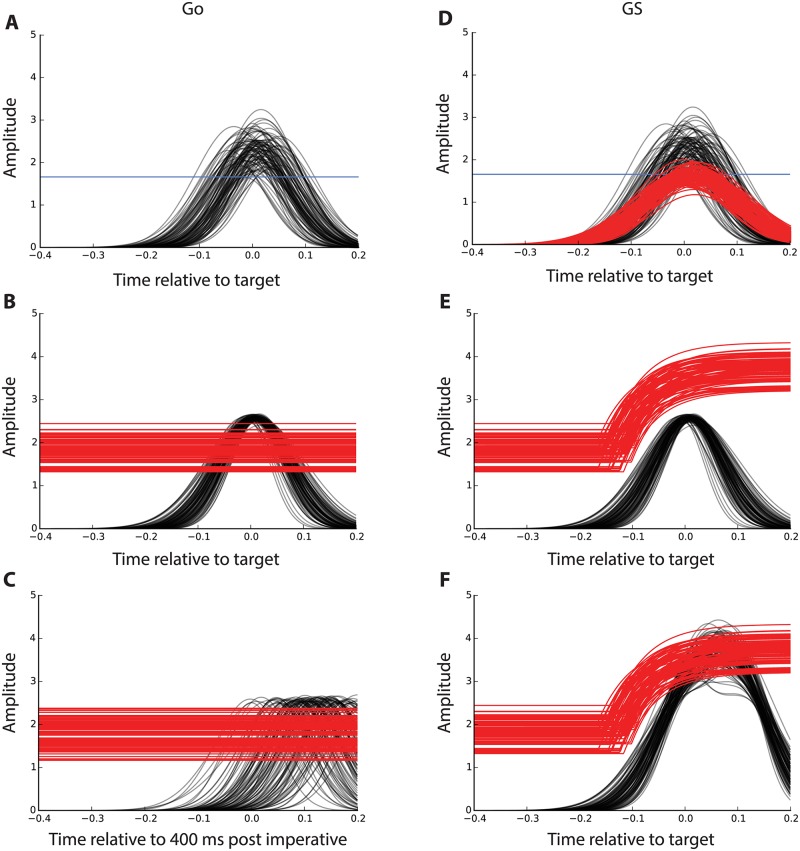
Model results for Go and GS trials. 100 simulated Go (A–C) and GS (D–F) trials using best fitting parameters produced by the activation threshold model (ATM; bottom four panels) and horse-race model (HRM; top two panels). A: The HRM captures MEP amplitude and EMG data from the anticipatory response inhibition (ARI) task. A single decision threshold is set at 1.659. B: The ATM is able to capture MEP and EMG data from the ARI task and necessitates a relatively narrow distribution of facilitatory drive. C: Using the ATM, bimanual Go reaction times in the stop-signal task are captured with a much wider distribution of facilitatory drive. D: The addition of inhibitory input to the HRM adequately captures modulation of corticomotor excitability but struggles to capture delayed EMG onset times and increased EMG onset rates that are empirically observed in the ARI task. E: The ATM demonstrates that facilitatory input for the Go response on ARI trials is unable to surpass the elevated activation threshold following nonselective inhibition of the bimanual response. F: A secondary facilitatory input is required to summate excitatory drive in the ATM to pass the elevated threshold and generate a unimanual left hand response. Red: inhibitory input (activation threshold); black: facilitatory input; blue: decision threshold.

Both models were able to reproduce the rise in MEP amplitudes and distribution of EMG onsets and offsets for Go trials. Best-fitting parameters and Chi-square goodness of fit are provided in Table 1. Fig 5 illustrates 100 simulated trials with the optimized parameters from the HRM (A) and ATM (B). The Akaike Information Criterion (AIC) was used to compare the HRM and ATM which have different degrees of freedom (7 versus 8 respectively). AIC was calculated as:

$$\begin{array}{c} AIC=\chi^{2}+2p+2p\left(p+1\right)/N-p-1 \end{array}$$ (6)

where *p* is number of model parameters and *N* is number of bins. Although the HRM (AIC = −8.38) yielded a slightly better fit than the ATM (AIC = −7.99) for Go trials, the ATM better captured the tight (small SD) distribution of onset times (Chi-square = 0.7 × 10^−6^) that is a characteristic feature of EMG and LT data in the ARI task (Fig 1A, right). Average predicted onset time for facilitation curves in the ATM was −157 ms and −159 ms in the HRM, consistent with the experimentally observed rise in CME 175 [7] to 150 ms [28] before the target.

**Table 1:** Output from the horse-race and activation threshold models for Go trials of the anticipatory response inhibition task.

Parameter	Estimated Value		Experimental Variable	*χ*^2^		Summed *χ*^2^	
	HRM	ATM		HRM	ATM	HRM	ATM
*a*_*facGo*_ (mean)	2.342	2.576	MEPs 150 ms	0.003	0.005	0.018	0.012
*a*_*facGo*_ (SD)	0.323	0.053	MEPs 125 ms	0.004	0.006		
*b*_*facGo*_ (mean)	0.007	0.006	MEPs 100 ms	0.007	0.001		
*b*_*facGo*_ (SD)	0.021	0.008	EMG onsets	0.003	0.7*x*10^−6^		
*c*_*facGo*_ (mean)	0.066	0.064	EMG offsets	0.001	0.1*x*10^−3^		
*c*_*facGo*_ (SD)	0.010	0.011					
*DecisionThresh*	1.659						
*Inhib*_*Go*_ (mean)		1.798					
*Inhib*_*Go*_ (SD)		0.246					
*t*_0_	−0.159	−0.157					

Estimated values for *b* and *t*_*o*_ reported in seconds relative to target. Experimental MEPs recorded at time in ms prior to target. HRM: horse-race model; ATM: activation threshold model; SD: standard deviation; MEP: motor evoked potential; EMG: electromyography.

### Partial trials

**Fig 6:**
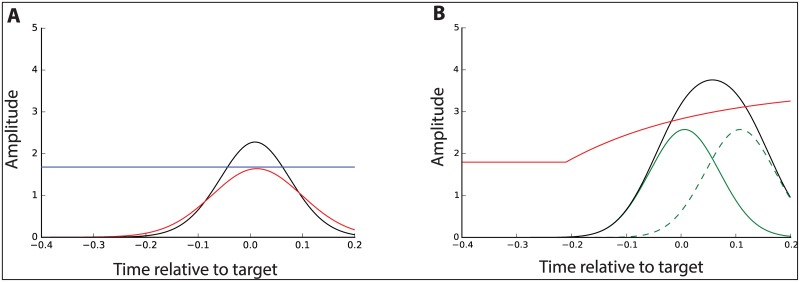
Average model simulations on GS trials. Average best-fitting facilitatory and inhibitory inputs simulated by the horse-race model (A) and activation threshold model (B). Facilitatory input surpassing the elevated activation threshold represents summation of the preplanned Go response (green) and reprogrammed unimanual movement (green dashed). Compare with model predictions in Fig 2C&2D. Red: inhibitory input (activation threshold); black: facilitatory input; green: facilitatory input for preplanned Go response; dashed green: facilitatory input for reprogrammed unimanual response; blue: decision threshold.

The optimized facilitation curves derived from Go trials were used for GS trials in both models. The best fitting parameters and Chi-square values for both models are provided in Table 2. 100 simulated trials with these parameters are shown in Fig 5D–5F and average best-fitting facilitatory and inhibitory curves are shown in Fig 6. AIC was again calculated and was −7.14 for the ATM and 96.92 for the HRM. While both models were able to reproduce the dip and subsequent rise in MEP amplitude for the responding finger on GS trials, the ATM was a better fit as indicated by asmaller AIC. black The HRM especially struggled to capture the distribution of EMG onset times, which contributed to thesubstantially larger AICvalue compared to the ATM.

**Table 2:** Output from the horse-race and activation threshold models for Partial trials of the anticipatory response inhibition task.

Parameter	Estimated Value		Experimental Variable	*χ*^2^		Summed *χ*^2^	
	HRM	ATM		HRM	ATM	HRM	ATM
*a*_*inhGS*_ (mean)	1.644		MEPs 75 ms	1.048	0.822	105.924	1.862
*a*_*inhGS*_ (SD)	0.177		MEPs 50 ms	0.892	0.568		
*b*_*inhGS*_ (mean)	0.015		MEPs 25 ms	0.080	0.003		
*b*_*inhGS*_ (SD)	0.008		EMG onsets	103.403	0.451		
*c*_*inhGS*_ (mean)	0.087		EMG rates	0.500	0.019		
*c*_*inhGS*_ (SD)	0.009						
*k*_*inh*_		1.887					
*τ*_*inh*_		0.060					
*step*_*t*_ (mean)		0.133					
*step*_*t*_ (SD)		0.018					
*b*_*facGSNew*_ (mean)		0.108					
*b*_*facGSNew*_ (SD)		0.002					
*t*_0_	−0.190	−0.055					

Estimated values for *b* and *t*_*o*_ reported in seconds relative to target. Experimental MEPs recorded at time in ms prior to target. HRM: horse-race model; ATM: activation threshold model; SD: standard deviation; MEP: motor evoked potential; EMG: electromyography.

#### HRM

Average predicted onset time (*t*_0_) for inhibition curves was −190 ms, which is 60 ms after presentation of the stop signal. This timing is consistent with the stop signal triggering the Stop process following a short delay for cue processing. Interestingly, the onset of inhibition is before the rise in facilitatory drive which occurs 159 ms before the target. However to capture the modulation of CME on these trials, the HRM demonstrates that the rise in inhibitory input is necessarily at a slower rate than facilitation and the facilitatory drive is able to reach the decision threshold first to generate the preplanned response in the left finger.

#### ATM: Bimanual inhibition

The temporal modulation of CME on GS trials was successfully recreated by increasing the activation threshold. *step*_*t*_ indicated that neural braking of the bimanual movement began on average 117 ms after the stop signal presented at −250 ms. Increased inhibition resulted in none of the simulations crossing threshold. Crucially, this model would incorrectly predict no response on successful GS trials. Initial facilitation curves that were suitable for simulating Go responses, were insufficient to cross the threshold on GS trials. Therefore an additional facilitatory input was needed to model the observed behaviour.

#### ATM: Unimanual facilitation

The ATM was able to capture EMG onsets of GS trials only with the inclusion of a second facilitation phase. The best fitting parameters and corresponding Chi-squares for the new facilitation curve are provided in Table 2. Fig 5F shows the combined facilitatory inputs passing the elevated activation threshold, thereby generating a response. The model also successfully characterized the elevated gain of the delayed partial response. Average predicted onset time (*t*_0_) for the second facilitation phase was −55 ms i.e., *after* nonselective inhibition at −75 ms. This second facilitatory input can be conceptualized as a newly programmed left hand response, subsequent to the cancellation of the default bimanual (Go) response.

### Go trials in the stop-signal task

The ATM successfully captured the distribution pattern of RTs from [13] on the SST, as indicated by very low Chi-square values. The best fitting values for *b*_*facGoSST*_ are reported in Table 3 and 100 simulated trials with these values are shown in Fig 5C. Fig 5B and 5C contrast the distribution of the Go responses between the ARI task and SST in the ATM framework.

**Table 3:** Model output for Go trials of the stop-signal task.

Trial	Parameter	Estimated Value	Experimental Variable	*χ*^2^
Go	*b*_*facGoSST*_ (mean)	0.129	RTs	0.001
	*b*_*facGoSST*_ (SD)	0.048		

Estimated values for *b* reported in seconds relative to target. SD: standard deviation; RT: reaction time.

## Discussion

A computational model of neuronal activity was put forward to explain the dynamics of RI and compared against a traditional horse-race model. Both the ATM and HRM were able to successfully fit neural excitability (MEP) and muscle activity (EMG) data observed empirically during execution of movement. However, as hypothesized, the ATM framework provided a better theory for the mechanisms underlying partial cancellation of movement by achieving a much closer fit with empirical findings on Partial trials. The ATM demonstrated that MEP suppression on Partial trials can be explained by nonselective inhibition. This inhibition raises an activation threshold to a level that cannot be reached by the preprepared Go response. In support of our hypothesis, additional facilitatory input was required to account for the delayed response executed at a higher gain. The ATM can also accommodate the much wider Go distribution of the SST compared to the ARI task. There are several reasons why the ATM provides a framework for understanding why partial RI may occur without delay on the SST, but not the ARI task.

The ATM was able to capture the tight temporal distribution of excitatory drive for the required bimanual responses of the ARI task, and also captured the broad distribution of responses in the SST. The anticipatory nature of the ARI task allows participants to perform Go trials very accurately [4, 29]. Participants begin each trial in this task intending to make a (default) bimanual response to stop both indicators at the stationary target. The standard deviation for the Go response for young adults is normally 20–30 ms [3] and was 8 ms for the EMG distribution in the ATM. The ATM provides computational evidence that it is not possible to produce the delayed response on Partial trials by merely sampling from the later part of the Go distribution (c.f. [15]). The tightly distributed execution process signifies that response delays in ARI tasks are immune to such “sampling bias”. This important difference means that the ARI task allows a more valid examination of the response delay during pure (reactive) RI than the SST.

Both the ATM and HRM present plausible mechanisms to reflect neural activity underlying the bimanual response. Average response asynchrony on Go trials for the ARI task is typically 3–8 ms [3, 6]. Regardless of muscle pairing, movement components are integrated together into a unitary response during movement preparation [7, 12], indicative of transient conceptual binding [30]. Functional coupling between hands is especially strong when homologous muscles are activated simultaneously [31, 32], as in the experimental data used in this study. The modulation of neural input captured by the two models on Go trials therefore accurately represents the generation of a unitary response which is anticipated as the default on every trial.

The HRM is able to simulate simple execution and inhibition of behaviour, but falls short compared to the ATM when trying to account for partial RI. The HRM produced a poor fit to the distribution of EMG onset times during GS trials, which is unsurprising given that the facilitation curves crossing the decision threshold on Go trials are necessarily the same on GS trials. The HRM therefore cannot account for the response delay routinely observed during partial RI. Of note, a necessary extension of the HRM is the divergent time course of inhibitory drive to the two fingers during Partial trials to explain the opposite behavioural outcomes (i.e. execution versus suppression). While facilitation wins the race for the responding finger, inhibition would presumably win the race for the cancelled finger. Importantly, the HRM does not support purely selective inhibition as inhibition is still present in both sides. However the neural substrate for inhibition might be subtly distinct between the two fingers. The current study cannot directly speak to whether there is a distinct latency for inhibitory drive between the two fingers during partial RI as no data was modelled for the cancelled movement. Although empirical findings of comparable MEP suppression at equivalent time points between the responding and cancelled fingers [7] would suggest the HRM explanation is unlikely. Overall, it seems the horse-race framework alone is not suitable for explaining the experimental findings during partial cancellation of movement.

On Partial trials, the stop cue introduces response conflict as the default response is no longer appropriate. The subsequent modulation of CME on these trials can be best explained by an ATM which dictates an increase in inhibition (i.e. an activation threshold). The increased threshold may explain the empirical suppression of CME that occurs 100–200 ms after the stop signal [6, 13, 28, 33–35]. In the presence of conflict, the presupplementary motor area (preSMA) activates the subthalamic nucleus via the nonselective inhibitory “hyperdirect” basal ganglia pathway to rapidly terminate the prepotent response [5, 36–39]. The functional role of this nonselective inhibition is similar to that described in the neural network model for an antisaccade task [36, 37]. Elevation of the threshold may represent the recruitment of an override mechanism during response conflict to suppress the default response and to enable executive control to take over. The empirical data show, and the model supports the contention, that nonselective inhibition suppresses all motor representations of the default (unitary) bimanual action.

Partial cancellation of multi-component movement involves task switching. [37] proposed that a raised threshold allows the switch from a prepotent to a controlled response to meet new task demands in the antisaccade task. On a successful antisaccade trial, an obvious switch in movement direction is seen. The ATM demonstrates that behaviour during partial movement cancellation can be explained by a comparable switching process, albeit less obvious: from a bimanual to unimanual response. The dorsolateral prefrontal cortex [40–42] and preSMA [38, 43–45] are activated during response conflict and facilitate a switch from a prepotent response to those requiring higher levels of cognitive control. The preSMA is activated during partial movement cancellation [5], potentially signalling the switch from a (prepotent) bimanual to (controlled) unimanual response.

A second facilitatory input is required in the ATM framework to meet Partial trial demands. A further argument against Partial responses being from the later part of the Go distribution (c.f. [15]) is that the neural activation for Go responses is unable to reach the elevated threshold, albeit according to the underlying assumptions of the model. An additional facilitatory process is recruited to add to and reshape excitatory drive. The second facilitatory component of the ATM may explain evidence of lateralized event related potentials 200–300 ms after a partial stop cue [46]. Recruitment of the additional facilitatory process signifies the selective preparation of the unimanual response. The presence of nonselective inhibition during the response preparation phase, as depicted in the ATM, fits with previous experimental findings. Task irrelevant muscles [47, 48] and the contralateral primary motor cortex [49] demonstrate CME suppression during selective movement preparation. Widespread CME suppression is observed even if response anticipation is not possible [50], as is the case on Partial trials following presentation of an unexpected cue. The working hypothesis is that nonselective inhibition during response preparation acts to reduce noise and thereby improve signal processing by enhancing the signal to noise ratio. The elevated threshold in the ATM following the stop cue may therefore represent both suppression of the default response to resolve conflict, as well as an increase in the signal to noise ratio to facilitate selective movement preparation. The additional excitatory drive therefore follows nonselective inhibition to selectively prepare and generate a new (unimanual) response rather than the partial continuation of a previous response.

The ATM can also successfully capture behaviour during complete cancellation of a multi-component response. If a second facilitatory drive is not present, the elevated threshold is not reached for either component and the ATM predicts no movement is generated. This behavioural outcome constitutes success on a trial requiring complete cancellation. Interestingly, the ATM can also recreate the experimental observation that participants (more often older adults) occasionally suppress their bimanual response following a partial stop cue, but are unable to generate a unimanual response within the time constraints. Absence of a unimanual response may result from the inability to uncouple bimanual components via rapid recruitment of neurons within the supplementary motor area [51, 52] and/or sufficiently summate facilitatory drive.

The simulations indicated that the main difference between the ARI and SST is in the distribution of Go responses. In SSTs like those employed in [13] and [15] (Fig 1B&1C), a choice paradigm results in right skewed response distributions [17, 27] with a larger standard deviation of up to 80 ms [11]. Across studies, average RTs are typically in the range of 300–600 ms. Strategic slowing can further delay RTs in the SST [14]. Wider, right skewed RT distributions could make it difficult to determine whether responses are genuinely delayed on Partial trials, or from a Go response that was slow to begin with [15]. We contend that the rate of EMG onset on Partial trials obtained in the SST may resolve this uncertainty. The novel prediction from the ATM is that EMG gain on Partial trials will not differ from Go trials for the SST, as it does for the ARI task, because the distribution of Go RTs negate the requirement for a second facilitatory drive.

The present study has some limitations. Firstly, the model assumptions outlined in the Methods dictate the characteristics and performance of the models. While the underlying assumptions were based on valid neurophysiological mechanisms, they should nonetheless be kept in mind when interpreting the model results. Secondly, there is always a risk of over-fitting optimization models to produce parameters that precisely match data from only one experiment, thus limiting the usefulness of the model. One solution is to do cross-validation, but this relies on having sufficient amounts of data given the stochastic nature of our model. We therefore opted for a purely descriptive approach in the first instance. Thirdly, there are risks associated with model identifiability. We can only conclude that our ATM is superior to the current HRM when attempting to explain neural processes and behaviour during partial RI. It remains to be determined how our model compares to a greater number of alternative explanations. For example, constraints may act directly on the facilitatory input i.e. decreased activation of facilitatory neurons, not increased activation of inhibitory circuits. Alternatively, facilitation might be monotonic and nonselective inhibition might demonstrate a phasic response through suppression of all motor output until the facilitatory command to the right hand is successfully cancelled, and then inhibition is released. However, [15] contend that such a “restart” model is inconsistent with the underlying assumptions of the traditional horse-race model as independence between the Go and Stop process is violated. Fourth, only the non-dominant hand was modelled in the present study, although the same mechanisms would be expected to apply to either hand responding on a Partial trial. Future use of the ATM could specifically investigate whether the model is a comparable fit to the dominant side when it is executed on Go and Partial trials. Finally, the ATM is unable to differentiate between neural inhibition occurring at cortical, subcortical and spinal levels. It represents a system-wide inhibitory process. However the system-wide approach can also be considered a strength; the ATM is able to capture the combined influence of all sources of inhibition within the motor system upstream of the alpha motoneuron pool.

Further investigations of the ATM might examine other and future experimental findings. For example, the neural mechanisms behind temporal modulation of CME during unsuccessful Partial trials. The ATM may also offer new testable hypotheses such as how RI is affected during healthy ageing or with dopaminergic dysregulation, such as that occurring with Parkinson’s disease.

## Supporting Information

S1 Code The Python code and data for the models are available at https://github.com/peppi107/Response_inhibition_models. (PY)
